# Impact of the COVID-19 Pandemic on Swedish Adolescents’ Mental Health, Psychosocial Functioning, Risk Behaviours, and Victimisation: Gender Differences and Implications

**DOI:** 10.3390/ijerph21050604

**Published:** 2024-05-09

**Authors:** Catrin Johansson, Britt Hedman Ahlström, Marijana Barac, Therese Berglund, Kourosh Bador, Nóra Kerekes

**Affiliations:** 1 Department of Health Sciences, University West, 461 86 Trollhättan, Swedennora.kerekes@hv.se (N.K.); 2 Centre for Holistic Psychiatry Research (CHoPy), 431 60 Mölndal, Sweden; 3 AGERA KBT, 411 38 Gothenburg, Sweden

**Keywords:** adolescents, COVID-19 pandemic, mental health, psychosocial functioning, risk behaviours, Sweden, victimisation

## Abstract

The COVID-19 pandemic has shown varying effects on adolescents’ mental health, psychosocial functioning, risk behaviours, and victimisation. This study aims to examine the changes reported by a sample of Swedish adolescents (*N* = 1607) at the end of the first year of the pandemic in relation to these factors. Data were collected with an electronic survey between September 2020 and February 2021, targeting upper-secondary high school students (aged 15–19 years). The results indicate a relatively low overall impact of the pandemic on Swedish upper-secondary school students, with notable gender differences. Compared to adolescent women, a higher percentage of adolescent men reported experiencing elevated levels of anxiety, depression, sleep disturbances, anger, and increased illicit drug use as consequences of the pandemic. In contrast, women demonstrated an increase in several salutogenic behaviours. Victimisation rates generally decreased during this period. These findings underscore the importance of heightened awareness among professionals within schools, social services, and healthcare settings regarding the distinct challenges encountered by a larger portion of adolescent men during the COVID-19 pandemic in Sweden.

## 1. Introduction

The COVID-19 pandemic and its societal consequences, without a doubt, significantly impacted adolescents’ transition into adulthood. The pandemic, coupled with its associated restrictions and changes, altered normal routines and disrupted many aspects of daily life for all. For example, Papadopoulos [1] and Foley et al. [2] investigated the impact of COVID-19 on family functioning and dynamics and on changes in children’s behaviours, revealing a negative influence. Besides changes in family functioning and dynamics for adolescents, one of the major impacts of the pandemic was the closure of schools and the start of distance education [3,4]. While these changes disrupted learning [5], they also limited in-person social interactions with peers [6]. During development, a child’s need for stable and safe interactions with family members shifts to include the need for social interactions with other adolescents [7]. Adolescence is a period of rapid changes in neurodevelopmental [8] and physical functioning, and therefore, it is also a time of increased vulnerability c.f. [9]. Pandemic-related disturbances to this social maturation process, coupled with the stress and uncertainty of the period, may have contributed to an increase in mental health problems such as anxiety, depression, and trauma symptoms among adolescents [10,11,12].

Previously reported risk factors for mental health problems during the pandemic include a history of mental health problems, a high number of stressful life events, an unstable family environment [13], and being a woman [14,15]. Past research, however, also identified protective factors for adolescents, such as effective social support and daily routines [3] and the presence of supporting family members, teachers, and other role models [16]. While the loss of social interaction with peers posed a risk factor, maintaining social connections with friends emerged as a protective factor [17], helping adolescents cope with the challenges and stresses of the pandemic. Physical activity, both individually and as a team activity, also had protective effects on adolescents’ mental health [18].

The pandemic’s interruption of adolescents’ social interactions with peers also altered their risk behaviours, such as substance use and antisocial behaviours, and influenced the form and frequency of their victimisation. The research on how adolescents changed their behaviours during the pandemic are conflicting, for example, concerning alcohol consumption [19] and cyberbullying, which was found to both increase [5,20] and decrease [21]. This emphasises the importance of focusing the well-known bio-psychosocial matrix on our behaviours and well-being [22].

The containment strategy of the Swedish government emphasized voluntary and individual accountability for the spread of the virus. Recommendations imposed by the government included social distancing, regular and cautious hand hygiene, isolation at home when experiencing any type of symptoms, avoiding physical contact with seniors, avoiding unnecessary travelling, and, if possible, working from home [23]. The rationale behind this approach was to balance the need to control the spread of the virus with minimizing the societal and economic disruptions associated with lockdowns [24]. As a consequence, even though Swedish adolescents were not affected by a lockdown, they were still required to limit social and physical contact with their peers and relatives. 

During the COVID-19 pandemic, there were surges in cases (described as waves), during which Swedish public health services enacted certain restrictions and recommendations. In Sweden’s first wave, March 2020 to July 2020 [25], upper-secondary school students were advised to switch to distance schooling [23]. Despite some limitations in social contact, it was still possible for adolescents to play sports. From 15 June 2020, distance schooling was withdrawn, and it was announced that education would return to normal during the next semester. By the end of the first wave, 80% of the Swedish population had adapted their everyday lives to decrease the risk of spreading the disease. The results of Kapetanovic et al.’s [26] study investigating changes in Swedish adolescents’ psychosocial functioning during this first wave (data collected from 8 June 2020 to 7 July 2020) showed that most adolescents followed government regulations. Additionally, most experienced less substance use and victimisation but poorer mental health. Adolescent girls and those studying through distance schooling were likelier to report negative changes in psychosocial functioning. This can be related to a study by Källmen and Hallgren [27], where adolescent women, in comparison to adolescent men, reported experiencing poorer mental health outcomes subsequent to exposure to COVID-19. But overall, the results in Kapetanocic et al. [26] study showed that most did not report any changes. The results also showed that some adolescents reported reduced peer interaction, parental conflict, and feeling a diminished sense of control over their lives. As mentioned above, while there are many research findings concerning the impact of COVID-19 on mental health and health conditions among adolescents during the first wave, there is far less research on the impact of the second wave.

In Sweden, the second wave of the pandemic began at the end of September 2020 and lasted until February 2021, leading to further recommendations. Students of all ages were advised to stay home if they were infected or lived with someone infected with COVID-19. In December 2020, upper-secondary school students had partial distance schooling. At the end of January 2021, Swedish public health services indicated that upper-secondary schools could withdraw partially from distance schooling [23]. In comparison to other countries around the world, Swedish restrictions on upper-secondary school students were relatively lenient. The present study aims to describe the changes Swedish adolescents reported at the end of the first year (the second wave) of the COVID-19 pandemic, considering their mental health, risk behaviours, psychosocial functioning, and victimisation, and compare the findings for women, men, and non-binary adolescents. During the interpretation of the results, our emphasis was on discerning the influence of pre-existing salutogenic behaviours in mitigating the impact of COVID-19 on the mental health and psychosocial well-being of adolescents. This analytical approach not only enriches our understanding but also contributes significantly to the domain of health promotion. Moreover, by scrutinizing the data through a biopsychosocial lens within the context of the pandemic, we aim to shed light on risk behaviours and victimisation, thereby augmenting knowledge in the fields of social psychiatry and criminology.

Based on studies from Sweden during the first wave [26] and from multinational data from the second wave [28], we hypothesise that Swedish adolescents adapted well to the circumstances of the COVID-19 pandemic, and therefore, the majority of them did not report negative changes in mental health, risk behaviours, psychosocial functioning, and victimisation. We also hypothesise that there were gender differences in how Swedish upper-secondary school students’ mental health, risk behaviours, psychosocial functioning, and victimisation changed during the COVID-19 pandemic. Our study may contribute to a deeper understanding of the impact of the second wave of the COVID-19 pandemic on adolescents’ psychosocial functioning, mental health, risk behaviours, and rates of victimisation in Sweden.

## 2. Materials and Methods

### 2.1. Study Design and Procedure

The international study Mental and Somatic Health without Borders (MeSHe) (https://meshe.se/) (accessed on 26 November 2023) employed a cross-sectional design and utilised an electronic survey to collect data. The data for the current study were collected in Sweden between September 2020 and February 2021 using this electronic survey. The survey consists of validated questionnaires that assess various aspects of adolescents’ mental and physical health as well as their risk behaviours. Specifically, the questionnaires in this study address the impact of COVID-19 and changes in adolescents’ behaviours, mental health, and experiences of victimisation during the COVID-19 period.

### 2.2. Study Population

Despite initial direct contact with the administrations of nearly all Swedish upper-secondary schools at the beginning of the fall semester in 2020, only 292 respondents were obtained, resulting in an unacceptably low response rate. To increase the sample size, the survey was made available on social media platforms during the Christmas holidays of 2020, specifically targeting 15–19-year-old upper-secondary school students (inclusion criteria). A total of 10,693 clicks were recorded on the link, with 59% from adolescent men and 41% from adolescent women, resulting in 1370 responses and a response rate of 12.81%. Responses were collected from individuals across all 21 counties in Sweden. Excluding those outside the age range of 15–19 years (n = 55), the final dataset consisted of 1607 responses, with a mean age of 17.12 (SD = 0.96), comprising 59% females, 40% males, and 1% identifying with other gender identities or with missing responses.

### 2.3. Instruments

The measures listed below have been previously employed in studies conducted with adolescent populations [26,28].

#### 2.3.1. COVID Impact

This item assessed the extent to which COVID-19 personally impacted the lives of adolescents. Participants provided their responses on a numeric analogue scale ranging from 0 (indicating minimal or no effect) to 10 (indicating significant and profound impact).

#### 2.3.2. Changes in Adolescents’ Behaviours during COVID-19

In this study, we opted to present the items in accordance with their contextual relevance.

(a) *Risk behaviours*, including the following: (1) consuming alcohol; (2) getting intoxicated by alcohol; (3) smoking cigarettes; (4) illicit drug use, including prescription drugs used for reasons other than prescribed; (5) arguing/fighting with a parent or parents; and (6) staying outside or being in the city without parents’ knowledge. Cronbach’s alpha for the risk behaviours in the present study was 0.69;
(b) *Norm-breaking behaviours*, consisting of the following: (1) stealing from shops, people, or from own or someone else’s home and (2) harassing someone on the internet using written language or uploaded pictures and/or videos;
(c) *Salutogenic approaches*, incorporating the following: (1) having the opportunity to be in control over one’s daily life, (2) keeping up with school projects and/or work, (3) spending time doing things that one did not have time to do before, (4) working out or exercising, (5) being outside and (for example) taking walks, (6) spending time with family and taking part in fun activities, (7) staying in contact with relatives and friends over the phone/internet, (8) staying connected with friends through social media or video games, and (9) meeting up with friends in real life. Cronbach’s alpha for the salutogenic approaches in the present study was 0.60.

Participants were provided with the following response options: “I didn’t before and haven’t started after”, “decreased a lot”, “decreased a little”, “no change”, “increased a little”, and “increased a lot”.

#### 2.3.3. Changes in Adolescents’ Mental Health

This questionnaire comprised 10 items and aimed to evaluate adolescents’ self-reported changes in sleep, stress, satisfaction, loneliness, involvement in society, and various affective states. Participants were asked to rate each item on a response scale consisting of four options: “I strongly disagree”, “I disagree”, “I agree”, and “I strongly agree”.

#### 2.3.4. Changes in Adolescents’ Victimisation

The frequency of changes in victimisation was evaluated using a subset of five items adapted from the Swedish Crime Survey [29]. These items included physical violence, threats, sexual harassment, and two items related to online victimisation. Participants rated the frequency of these experiences on a 5-point scale, with response options ranging from “decreased a lot” to “increased a lot”.

### 2.4. Statistical Analysis

All analyses were conducted using IBM SPSS Statistics version 28. Descriptive statistics, including mean (M), median (Md), standard deviation (SD), and frequencies (%), were utilised to summarise the data. Chi-square tests and odds ratios were employed to compare the prevalence and odds associated with changes in mental health, risk behaviours, psychosocial functioning, and victimisation across genders. The distribution of responses for the COVID-19 impact item was assessed using the Shapiro–Wilk test, which revealed a significant deviation (*p* < 0.001) from normality. Therefore, differences in this item between genders were examined using the non-parametric Kruskal–Wallis test. We utilised the Kruskal–Wallis test since there were more than two genders (men, women, and non-binary), rendering bivariate analysis (such as the Mann–Whitney U test) inappropriate. While the Kruskal–Wallis test is typically used for three or more groups, it can also be applied to compare two groups when the data do not follow a normal distribution (https://statistics.laerd.com/spss-tutorials/kruskal-wallis-h-test-using-spss-statistics.php) (accessed on 1 May 2024). This is because the Kruskal–Wallis test is based on ranks rather than actual data values, making it robust to deviations from normality. Therefore, even when comparing two groups with non-normally distributed data, the Kruskal–Wallis test can still provide valid results. The significance level was set at *p* < 0.05.

## 3. Results

### 3.1. Impact of the COVID-19 Pandemic on Swedish Adolescents

**Figure 1:**
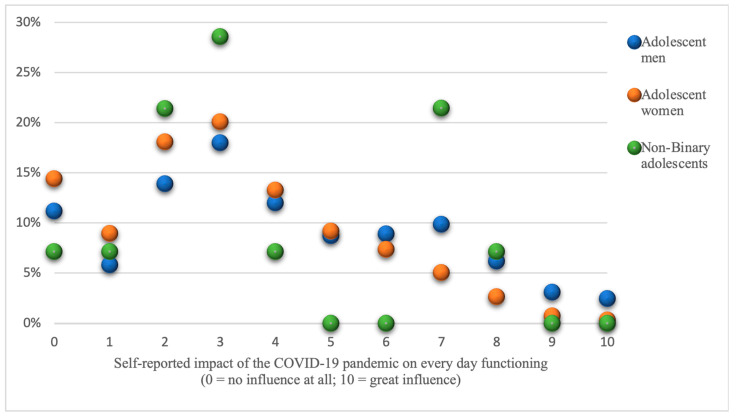
The proportions of adolescents reporting varying degrees of impact from the COVID-19 pandemic on their daily lives and functioning, presented by gender.

Figure 1 illustrates the distribution of responses from Swedish adolescents regarding the impact of the COVID-19 pandemic on their everyday lives, measured on a scale from 0 to 10. The proportions of men, women, and non-binary-gendered adolescents reporting their perceived impact are displayed.

A total of 1584 participants (98.5% of the study population) provided responses to this question. Among them, there were 618 men, 951 women, and 14 non-binary-gendered adolescents. Due to the limited number of non-binary-gendered respondents, statistical analysis could not be conducted for this group. The median score for the entire study population was three (Md = 3), indicating a moderate level of perceived impact.

Significant gender differences were observed, with students who identify as men reporting a significantly higher impact of COVID-19 on their everyday lives compared to women students (Md = 4 and 3; M = 4.03 and 3.14; SD = 2.49 and 2.21, respectively; *p* < 0.001) (Figure 1).

### 3.2. Changes in Swedish Adolescents’ Behaviour during the COVID-19 Pandemic

The questionnaire used in this study aimed to capture any changes in adolescents’ behaviours during the COVID-19 pandemic and to determine the direction of those changes. It also assessed whether adolescents had engaged in specific behaviours before the pandemic.

Responses on the absence of these behaviours provide insight into the overall behaviour patterns of Swedish adolescents regardless of the COVID-19 pandemic. By utilising these data, we were able to calculate gender-dependent odds ratios for specific defined behaviours.

Regarding risk behaviours, no gender differences were observed in the proportion of adolescents who reported not smoking (approximately 80%), not consuming alcohol (around 50%), and not getting intoxicated by alcohol (approximately 40%). However, significant differences (*p* < 0.001) were noted in the frequency of illicit drug use and staying outside/being in the city without parental knowledge. Adolescent men were 41% likelier to indicate occasional engagement in these behaviours compared to women. On the other hand, women exhibited an increased risk of arguing with their parents even before the COVID-19 outbreak (Table 1).

In the context of norm-breaking behaviours, there were no gender differences found in the proportion of adolescent men and women who indicated that they had never stolen from shops, people, or their own home (over 90%). However, a slightly higher proportion of adolescent women (98%) reported that they had never harassed someone on the internet using written language or uploaded pictures and/or videos compared to adolescent men (96%). This indicates that the male gender was associated with a significant (*p =* 0.025; 43%) generally increased risk (not as a consequence of the COVID-19 pandemic) of engaging in harassment (Table 1).

In terms of salutogenic behaviours, the proportion of adolescents reporting that they had never utilised these approaches was relatively low, ranging from 2% to 13%. Overall, Swedish adolescents indicated a high level of engagement in these behaviours as a general trend. There were, however, significant differences (*p* ranging between 0.003 and <0.001) between genders in some of these behaviours. Adolescents men were 36% less likely than women to report that they had never been outside, 34% less likely than women to report that they had never used phone/internet to keep in contact with relatives and friends, and 39% less likely than women to report that they never met with friends in real life. In other words, these behaviours were more often present in adolescent women’ behaviour even before the COVID-19 outbreak. Conversely, a higher proportion of female students reported that they had never used social media or video games to stay in contact with friends (14% vs. 8% of men), indicating that this type of behaviour is generally more associated with men (Table 1).

Table 2 describes the proportion of adolescents who reported changes (either a decrease or increase) compared to those who reported no change in these behaviours (including responses indicating that they had never engaged in the behaviour or that it remained unchanged during the COVID-19 pandemic) (Table 2).

For most behaviours, there were no significant differences in the proportion of young men and women who reported changes during the COVID-19 pandemic. However, adolescent women reported a significantly higher proportion of changes in four behaviours—spending quality time with family, having the opportunity to control everyday life, frequency of arguments with parents, and frequency of meeting friends in real life. On the other hand, men reported a significantly higher proportion of changes in two behaviours—illicit drug use and staying in contact with friends via social media (Table 2).

**Figure 2:**
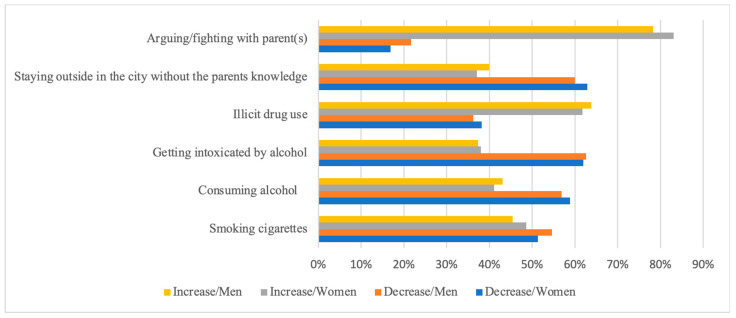
The proportion of adolescent men and women who reported decreased or increased risk behaviours during the COVID-19 pandemic.

Risk behaviours generally decreased in a higher proportion among both adolescent men and women, with the exception that a greater proportion of men (*p* = 0.83) reported increased illicit drug use, and a larger proportion of women (*p* = 0.15) reported an increase in the frequency of arguing with their parents (Figure 2).

**Figure 3:**
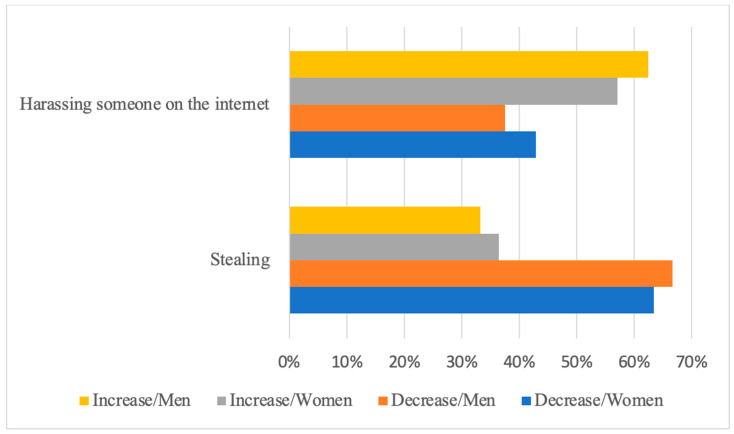
Decrease and increase of norm-breaking behaviours of adolescent men and women during the COVID-19 pandemic.

No significant differences were found in the proportion of adolescent women and men who reported an increase or decrease in either of the two norm-breaking behaviours. Both young men and women reported similar proportions, with the majority indicating a decrease in incidents of stealing while reporting an increase in incidents of harassing someone over the internet (Figure 3).

**Figure 4:**
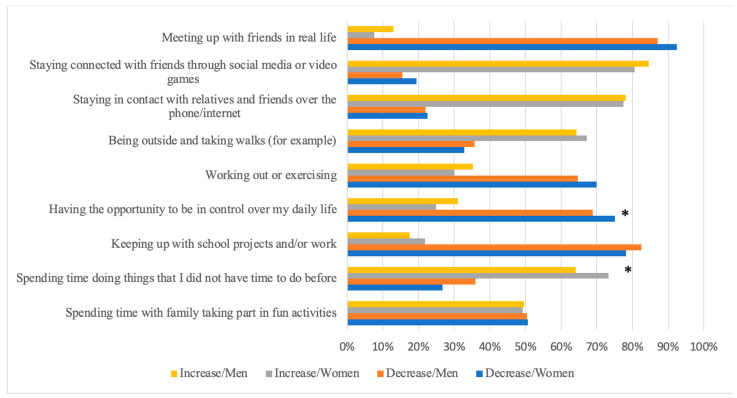
Decrease and increase in salutogenic approaches of adolescent men and women during the COVID-19 pandemic (* = significant difference).

Significant gender differences were found within reported changes in salutogenic behavioural approaches. Specifically, 73% of young women reported an increase in “spending time doing things that I did not have time to do before” compared to 64% of young men (*p* = 0.005). Additionally, 75% of adolescent women indicated a decrease in having a sense of control over their daily life, while 68% of men reported a decrease in this aspect (*p* = 0.029). Furthermore, a significantly higher proportion of adolescent women (*p* = 0.002) reported a decrease in meeting up with friends in real life (Figure 4).

In general, among those who reported any changes, a higher proportion indicated a decrease in their ability to keep up with schoolwork, having control over their lives, and engaging in exercise. On the other hand, a higher proportion reported an increase in the frequency of spending time doing things that they did not have time to do before (Figure 4).

### 3.3. Changes in Swedish Adolescents’ Mental Health during COVID-19 Pandemic

**Figure 5:**
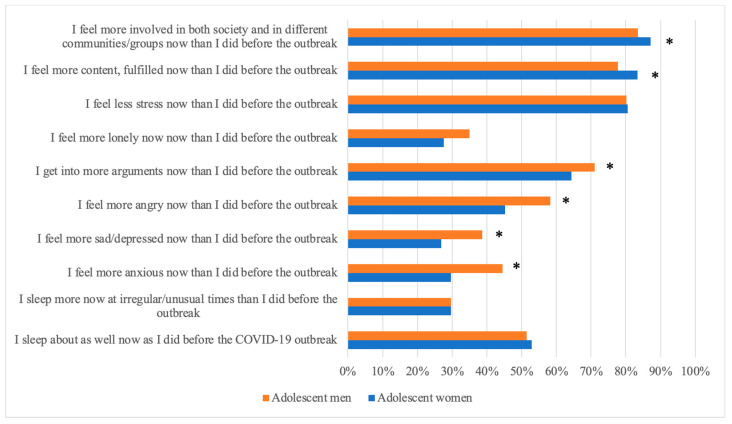
Reported changes in Swedish adolescents’ mental health, by gender, during the COVID-19 pandemic. Before implies prior to the outbreak of the COVID-19 pandemic (* = significant difference).

Several significant differences were observed in the proportion of adolescent women and men, indicating negative changes in their mental health during COVID-19 (Figure 5). A significantly higher proportion of men reported increased anxiety (44% of men, 30% of women; *p* ≤ 0.001), increased depression (39% of men, 27% of women; *p* ≤ 0.001), increased anger (58% of men, 45% of women; *p* ≤ 0.001), increased frequency of conflicts (71% of men, 64% of women; *p* = 0.006), and feelings of loneliness (35% of men, 28% of women; *p* = 0.002) during COVID-19 compared to before the outbreak. On the other hand, a higher proportion of adolescent women reported feeling more content or fulfilled (83% of women, 79% of men; *p* = 0.043) and being more active in society during the COVID-19 pandemic (87% of women, 84% of men; *p* = 0.043) compared to before the outbreak (Figure 5).

### 3.4. Changes in Experiencing Victimisation during the COVID-19 Pandemic

**Figure 6:**
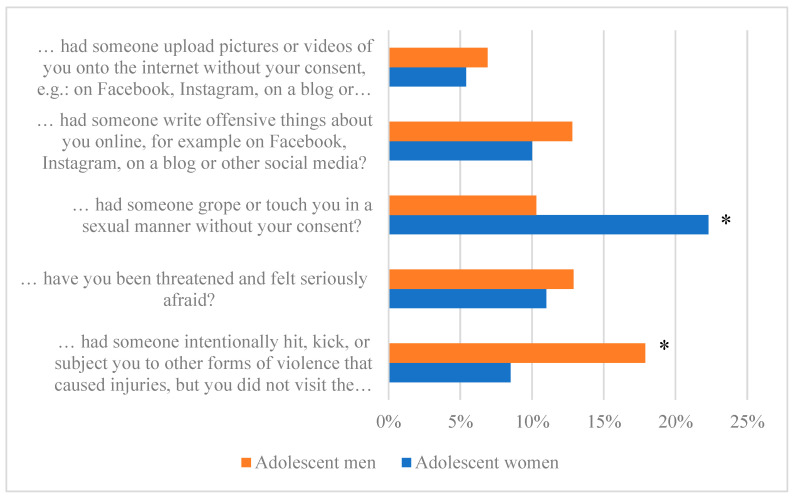
The proportion of adolescents by gender experiencing victimisation during COVID-19 (* = significant difference).

Most Swedish adolescent (77–95% of men and 82–93% of women) respondents did not experience any form of victimisation during the COVID-19 pandemic. A significantly higher proportion of adolescent men than women (18% of men, 9% of women; *p* = 0.001) reported being intentionally hit, kicked, or subjected to other forms of violence that caused injuries but did require them to visit the hospital. On the other hand, a significantly higher proportion of adolescent women than men (22% of women, 10% of men; *p* = 0.001) indicated that someone groped or touched them in a sexual manner without their consent (Figure 6).

**Figure 7:**
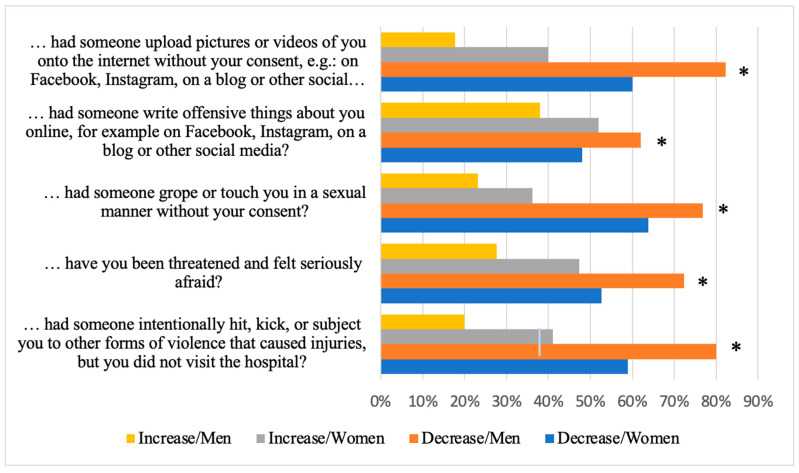
Decrease and increase in victimisation by gender during the COVID-19 pandemic (* = significant difference).

For all items assessing changes in experiencing victimisation during the COVID-19 pandemic, a higher proportion of adolescents reported a decrease rather than an increase in different types of victimisation. There was a significantly higher proportion of adolescent men who reported decreased victimisation compared to women (*p* ranging between <0.001 and 0.035) (Figure 7).

## 4. Discussion

The findings of this study reveal that the pre-existing salutogenic behaviours exhibited by Swedish adolescents played a significant role in mitigating the impact of COVID-19 on their psychosocial functioning and mental health. It is evident that the majority of participants actively engaged in various life events by maintaining social connections with friends and family, continuing to engage in physical activity, and staying on track with school projects, among other activities. These findings correspond to the research conducted by de Zarate et al. [30], who explored the association between physical activity, personal social contact, and well-being among adolescents during the COVID-19 pandemic. They observed a positive correlation between physical activity, personal social contyeact, and the well-being of young individuals. Thus, the salutogenic behaviours mentioned in the present study can be interpreted as protective factors. In a similar vein, Fan et al. [31] emphasised the significance of employing effective coping strategies to mitigate the adverse effects of the pandemic on daily life.

In this section, the study’s results concerning adolescents’ everyday lives and psychosocial functioning are discussed in relation to factors supporting health, well-being, and resilience. Moreover, the study’s findings pertaining to Swedish adolescents’ experiences of mental health, risk behaviour, and victimisation in the context of the COVID-19 pandemic are explored, and gender differences and their implications are also discussed.

### 4.1. The Impact of the COVID-19 Pandemic on Adolescents’ Everyday Lives

The present study reveals that Swedish upper-secondary school students reported a generally low impact of the COVID-19 pandemic on their everyday lives. This data file was part of a multinational analysis comparing the impact of COVID-19 across different countries [28], where it was noted that Swedish adolescents reported a lower impact compared to adolescents from other countries. One possible explanation could be that restrictions in Sweden were less severe than in other countries. Another possible explanation for this phenomenon is that their pre-existing salutogenic behaviours are rooted in positive sociocultural and socioeconomic factors. Such factors may have equipped Swedish adolescents with strong adaptability, contributing to their reports of being less negatively impacted by the COVID-19 pandemic than adolescents from other countries. Masten [32] described how all individuals possess the capacity to adapt, but some exhibit greater resilience due to their positive relationships with family, peers, and even teachers, which act as protective factors. Sweden is a highly developed country with a high standard of living. For example, the Organisation for Economic Cooperation and Development (OECD) [33] notes that Swedes report higher-than-average life satisfaction and have stronger social networks when compared to the OECD average. This is important to consider, as it paints a picture of a nation of content individuals who can rely on their relationships during challenging times, showing why the resilience described by Masten [32] is more apparent among Swedish youth. Another relevant study that explored psychological resilience among slightly older adolescents (18–24 years) was conducted by Renati et al. [34] in Italy. Their findings suggest that psychological resilience serves as a mitigating factor for potential emotional disturbances arising from adverse circumstances, such as pandemics.

Interestingly, in a multinational sample, a higher proportion of adolescent men compared to women or non-binary-gendered students reported a lower impact of COVID-19 on their lives [28]. However, in the present Swedish sample, adolescent men reported a significantly higher impact of COVID-19 compared to women. This finding suggests that the stronger impact on adolescent men in the Swedish context may be attributed to culturally specific gender differences in how adolescents coped and behaved during the first year of the pandemic.

### 4.2. Changes in Psychosocial Functioning during the COVID-19 Pandemic

Only a small proportion of Swedish adolescents reported a lack of salutogenic approaches in their lives, which partially explains the low impact of the pandemic on their everyday lives, as reported above. Among those who reported changes in suggested salutogenic approaches, a higher proportion indicated a decreased ability to keep up with schoolwork, feel control over their lives, and participate in exercise. However, a higher proportion also reported engaging more frequently in activities they did not have time for before. A similar proportion of both genders reported an increased frequency of participating in outdoor activities, such as taking walks. While slightly more young women than men reported feeling more content and active in society during the COVID-19 pandemic, more adolescent men reported increased internet use. Internet usage generally differs between genders, however, as noted by Sun et al. [35]. Although more adolescent men reported increased internet usage, they may use it in different ways. Young men were 57% likelier to use the internet to stay connected with friends through social media or video games, a behaviour that further increased during the pandemic for both genders.

Approximately 80% of both genders increased their online contact with relatives and friends, given the circumstances and restrictions in Sweden that focused on limiting in-person contact. This could be partially viewed as a potentially problematic use of the internet. Orhon et al. [36] found that problematic internet use among adolescents during the pandemic was associated with poorer sleep quality. Lower psychosocial functioning, such as a lack of physical activity, reduced academic performance, and problematic relationships with parents, were identified as predictors. However, for adolescents, using the internet as a means to stay connected with others was also an important tool to avoid social isolation, maintain social networks, and protect their mental health, as supported by previous studies [37].

### 4.3. Adolescents and Family Time during the COVID-19 Pandemic

It is common for young individuals to seek independence and distance themselves from their parents [38]. However, the COVID-19 restrictions created a situation in which parents and adolescents were confined together at home throughout the day. Interestingly, our findings indicate that this increased family time had a more noticeable impact on adolescent women, leading to an increased frequency of arguments and conflicts.

It is noteworthy that only one-fifth of the women participants in our study reported never having arguments with their parents, whereas almost one-third of the men participants reported the same. Despite this difference, both adolescent men and women spent a similar amount of time with their parents, and the proportion of adolescents experiencing changes (either a decrease or an increase in their behaviour) did not differ significantly between genders. These findings suggest that the majority of adolescents in our study had healthy relationships with their parents, which can be considered a protective factor against the impact of the pandemic. This can also be correlated with the findings of Herke et al. [39], who posited that a favourable familial environment is significantly related to positive health and well-being outcomes among children and adolescents. To further extend the discussion concerning adolescents´ familial experiences during the COVID-19 pandemic, the findings show that while young women reported significantly more arguments with their parents during the COVID-19 pandemic, they also reported significantly less frequently than men that they had stayed outside without their parents’ knowledge. This suggests that adolescent women are likelier to ask for permission and engage in arguments with their parents, while men tend to act more independently.

In a study by Magson et al. [17], an increase in conflicts between adolescents and their parents was found to be correlated with a decrease in life satisfaction during the COVID-19 pandemic. In line with this, our study revealed that a significantly higher proportion of women students reported a decrease in their sense of control over their daily lives, which may have contributed to a decrease in life satisfaction. This finding aligns with the results of the Moksnes et al. [40] study, which demonstrated that adolescent women exhibited a stronger negative association between interpersonal and school-related stressors and life satisfaction compared to adolescent men. However, it is important to note that throughout the 10-year period of the Moksnes et al. [40] study, life satisfaction remained consistently at high and stable levels, as measured in three cross-sectional assessments conducted in 2011, 2016, and 2022.

### 4.4. Changes in Mental Health during the COVID-19 Pandemic

The present study revealed that more Swedish adolescent men reported an increase in feelings of anxiety, depression, loneliness, anger, and involvement in conflicts (not limited to conflicts with their parents) compared to their women counterparts. Additionally, we observed that over 80% of those who reported worsened mental health also reported an increase in illicit drug use. This finding is noteworthy because a previous review [41] and a study focused on the same population [28] in a multinational context showed a higher increase in mental health issues among young women. In the context of Swedish adolescents, several studies demonstrated that adolescent women reported experiencing poorer mental health outcomes such as headaches, depression, feeling fear, stomach problems, difficulty sleeping, poor appetite, and increased anxiety and worry levels after exposure to COVID-19 compared to adolescent men [27,42,43].

The conflicting findings between these Swedish studies are fascinating yet challenging to explain. One possible explanation could be that the different studies [42,43] examined only limited aspects of mental health, focusing on anxiety and various worry themes as well as depression and somatic complaints [27]. In contrast, the current study conceptualises mental health in a broader sense, encompassing symptoms of anxiety, depression, loneliness, anger, and involvement in conflict. This comprehensive approach may distribute the load of negative effects across different factors, potentially leading to a reported decreased intensity compared to when it is expressed solely through one individual concept.

The different findings from studies with multinational samples may be attributed to the unique socio-cultural position of Swedish adolescent women. A previous study on Swedish adolescents’ psychological distress levels indicated that adolescent women exhibited a significantly stronger decrease in mental health in response to negative psychosocial factors within their families compared to adolescent men [44]. Combined with our finding that more adolescent women reported an increased number of conflicts with their families, while more students who identify as men reported decreased mental health as a consequence of the COVID-19 pandemic, this may suggest that changes at the micro-environmental level (such as within the family) may have a stronger impact on women, while changes at the macro-environmental level (such as a pandemic) may more strongly affect men. This underscores the significance of gender differences in adolescents’ ability to cope with their surrounding environment and possible protective factors during the COVID-19 pandemic.

### 4.5. Risk Behaviours before and during the COVID-19 Pandemic

One of the most important findings of the present study is that the majority of Swedish adolescents (93% of women and 87% of men) reported never using illicit drugs. Among the small percentage who used illicit drugs (13% of men and 7% of women), a significant proportion (84% of men and 71% of women) reported changes in the frequency of use. Approximately 60% of those (similar proportion in both genders) who reported changes also reported an increase in illicit drug use, representing almost 7% of men and 3% of women in the study population. The level of substance use (alcohol and drug) and the corresponding changes during the COVID-19 pandemic within the same sample as our study were extensively examined and quantified using specific measures of alcohol and drug use (AUDIT and DUDIT) in a recent publication by Sfendla et al. [45]. 

The increase in drug use among our study population contradicts the information about a general decrease in drug use from another developed country, Spain [46]. It was previously noted that adolescent men tend to self-medicate with illicit drugs or alcohol rather than seeking help when facing various mental health issues [47]. The higher proportion of adolescent men in our study engaging in increased illicit drug use during the COVID-19 pandemic may be both a consequence and a contributing factor to their reported higher rates of worsened mental health, including increased anxiety, depression, sleep problems, and anger. This pattern of increased illicit drug use among young men was also observed in the multinational sample [28]. The significant prevalence of illicit drug use as a way of coping among Swedish adolescents, especially when compared to other countries, highlights the ongoing need for heightened awareness among their social networks, including family and friends as well as various organisations (such as schools, sports clubs, social services, and health services), to effectively recognise and respond to signs of addiction, self-harm, and norm-breaking behaviours even years after the COVID-19 pandemic.

Regarding alcohol use, a similar proportion (about 40%) of adolescents women and men reported never using alcohol, and approximately 50% of both genders reported never getting intoxicated. Both genders, in similar ratios, reported decreasing their alcohol consumption and instances of getting intoxicated more than increasing them. Our finding that about 60% of adolescents used alcohol prior to the pandemic and that its use decreased during the COVID-19 pandemic aligns with a study conducted among Catalan adolescents (14–18 years old) [46]. A study by Vallentin-Holbech [48] observed a decline in alcohol consumption among Danish students during the second wave of the COVID-19 pandemic, coinciding with increased restrictions. Decreased alcohol use was attributed to reduced opportunities to purchase alcohol and drink in public spaces and the absence of social gatherings and parties, where alcohol is typically more accessible. The relatively high proportion of Swedish adolescents consuming alcohol reflects the cultural norms and expectations in Western countries [49].

In our study, young men were 43% likelier than women to report engaging in online harassment as perpetrators, although this behaviour was reported by a very small proportion (4%) of the study population. The reported increased time spent on the internet provides more opportunities for harassment, which could explain the corresponding increase in this item. It is possible that young women adhere more to gender-appropriate behaviour norms, leading to positive online experiences, while men may have negative experiences [35]. This could also explain why more adolescent men reported experiencing increased victimisation and harassment online.

### 4.6. The Frequency of Victimisation before and during the COVID-19 Pandemic

The majority of Swedish adolescents in our study (ranging from 77% to 95% of men and 82% to 93% of women) reported not experiencing victimisation during the COVID-19 pandemic. However, among those who did report being victimised, a notable gender-specific pattern emerged. Significantly more young men reported physical assault, while significantly more young women reported sexual assault, aligning with well-documented patterns of victimisation. Westlund and Öberg [50] highlighted that crimes against another person, including robbery, violence, and sexual offences, are common among youth, additionally aligning with our findings. This consistency can be attributed to the absence of a total lockdown in Sweden, which allowed for social contact, albeit somewhat limited. Additionally, Axell [51] revealed that young women aged 16–24 are likelier to experience intimate partner violence compared to women over 25 years, with one in five reporting various forms of victimisation by their partner or ex-partner. However, in the context of cyberbullying involving children and adolescents during the COVID-19 pandemic, Sorrentino et al. [52] conducted a systematic review and found an increase in the prevalence of cyberbullying in several Asian countries and Australia, while Western countries experienced a decline. Interestingly, our study indicated a higher proportion of adolescents reporting a decrease rather than an increase in victimisation, with significantly more men reporting a decreased frequency compared to women. This overall decrease in victimisation could potentially be attributed to the increased time spent in online schooling, as previous research has indicated that most crimes against children and adolescents occur within school environments [53]. Furthermore, our findings suggested that adolescent men experienced a greater decrease in all victimisation indicators, possibly indicating that adolescent men are both perpetrators and victims in environments outside of their homes, such as schools, of which they were partly deprived during the COVID-19 pandemic.

## 5. Strengths and Limitations

The present study displays a notable strength in its extensive and diverse study population, encompassing individuals from all counties of Sweden. However, it is important to acknowledge that this population is not fully representative due to the non-probabilistic sampling method employed. One of the strengths of this study is its adherence to ethical standards, having been controlled, adapted, and approved by the Swedish Ethical Review Authority. In Sweden, adolescents aged 15 and above are not required to obtain parental consent to participate in research. All adolescents involved in this study were informed that participation was voluntary and anonymous. Informed consent was obtained electronically at the beginning of the survey. Furthermore, at the end of the survey, respondents were directed to a homepage containing information about national resources and links to support organizations. An additional strength lies in the use of anonymous self-reports, facilitating more open disclosure of norm-breaking behaviours or experiences of victimisation without concerns about shame or consequences. Nevertheless, this approach also poses a limitation, as it lacks the ability for objective verification through official records or clinical data. Furthermore, retrospective perceptions of changes may not perfectly align with observations that could have been made in a longitudinal prospective study, underscoring another limitation. Additionally, the cross-sectional design restricts the ability to draw conclusions about causality or developmental trajectories. Our decision not to calculate the sample size before conducting an observational epidemiological study is influenced by the complexity of the research question and the lack of prior data on the subject (ongoing pandemic). Another limitation is that the COVID-19 Impact question and the Changes in Adolescents’ Behaviors during COVID-19 questionnaire have not been validated. In the present study, we categorized the items based on contextual relevance, which may introduce some subjectivity into the analysis. Of particular concern are the gaps in data collection regarding potential shifts and constraints in social, educational, and medical services, which could impact the mental health of adolescents. Although socioeconomic status was not directly assessed, it is worth noting that the participants generally had stable internet access, which is consistent across urban and rural areas in Sweden. 

## 6. Conclusions

Our study provides valuable insights into the impact of the COVID-19 pandemic on psychosocial functioning, risk behaviours, and mental health among Swedish adolescents. One notable finding is that the pandemic appears to have had only a modest effect on Swedish upper-secondary school students. Particularly relevant is the higher proportion of adolescent men reporting a deterioration in their mental health as a consequence of the pandemic. Conversely, adolescent women demonstrated an increase in several salutogenic behaviours, such as spending time with family and taking part in fun activities and meeting up with friends in real life, while men displayed an increase in negative behaviours, such as engaging in illicit drug use. However, it is important to note that these response patterns were observed for only a limited number of selected questions, where the proportion of respondents represented a small fraction of the overall study population.

## 7. Practical Implications and Future Research

At the time of writing, it has been over four years since the outbreak of the COVID-19 pandemic, a global event that has had a profound impact on the world. It is crucial to carry the learning gained from the experiences of the pandemic into the future. To effectively understand and address the consequences of such events, especially their impact on younger generations, it is essential to build a comprehensive sociocultural database of evidence-based studies in this field.

Our study focuses on a sample of Swedish adolescents, providing insight into how they adapted to the circumstances of the COVID-19 pandemic as well as their resiliency and ability to cope. The results highlight the critical need for early intervention and engagement, especially from educational institutions, to tackle mental health issues and prevent further decline among adolescents. One of the most significant findings of our study was the association between worsened mental health and increased illicit drug use among adolescent men. Recommended interventions to address gender-based disparities in mental health and illicit drug use among adolescents include establishing peer support groups for adolescent men, where they can openly discuss their concerns and experiences in a safe environment, and creating mentorship programs that pair them with positive male role models for guidance and support. Raising awareness about how young men express and experience poor mental health is essential for timely support and intervention. Community outreach initiatives targeted at adolescent men and their families can play a vital role in this regard. Further studies should focus on representative samples of adolescents in Sweden and examine not only the immediate but also the long-term effects of the pandemic, which spanned approximately two years and had a significant impact on various aspects of adolescent development. It is crucial to follow-up on the well-being and experiences of individuals who reported worsened mental health, increased victimisation, and increased illicit drug use as a result of the pandemic. Additionally, researchers could explore the support and assistance that Swedish organisations involved with youth could offer in developing effective strategies for addressing the consequences of the pandemic and promoting positive mental health outcomes among adolescents. Longitudinal studies tracking changes over time or focusing on interventions to mitigate negative impacts would also be particularly relevant. Ultimately, this study holds significance for policymakers as they deliberate on the potential duration and severity of future restrictions and the resulting consequences. This is particularly relevant due to the fact that Sweden’s restrictions during the COVID-19 pandemic were relatively more lenient or deviated from those imposed in other countries.

## Figures and Tables

**Table 1:** The proportion of adolescent men and women reporting the absence of a specific type of behaviour before and after the outbreak of the COVID-19 pandemic and the odds ratio for adolescent men to have certain behaviours.

		“I Didn’t Do It before and Haven’t Started during or after COVID-19”			Odds Ratio for Adolescent Men of Having Certain Defined Behaviours in Comparison to Women	
		Women %	Men %	*p*	OR	CI Lower/Upper
**Risk** **behaviours**	Smoking cigarettes	82.10	80.10	0.33	1.08	0.93/1.26
	Consuming alcohol	41.00	43.40	0.38	0.94	0.83/1.073
	Getting intoxicated by alcohol	50.20	49.50	0.78	1.02	0.90/1.15
	Illicit drug use, including the use of prescription drugs for reasons other than prescribed	92.70	87.20	<0.001	1.41	1.19/1.66
	Staying outside/being in the city without parents’ knowledge	53.50	44.50	<0.001	1.25	1.1/1.42
	Arguing/fighting with a parent or parents	20.80	31.10	<0.001	0.74	0.65/0.84
**Norm-breaking** **behaviours**	Stealing from shops/people or from own or someone else’s home	92.70	91.40	0.35	1.11	0.90/1.37
	Harassing someone on the internet using written language or uploaded pictures and/or videos	98.00	96.10	0.025	1.43	1.09/1.88
**Salutogenic** **approach**	Spending time with family and taking part in fun activities	3.80	3.80	0.97	0.99	0.72/1.38
	Spending time doing things that one did not have time to do before	4.90	3.90	0.39	1.16	0.82/1.63
	Keeping up with school projects and/or work	1.90	2.40	0.53	0.88	0.59/1.30
	Having the opportunity to be in control over one’s daily life	2.00	1.70	0.70	1.10	0.67/1.81
	Working out or exercising	11.70	13.40	0.36	0.91	0.76/1.10
	Being outside and taking walks (for example)	2.60	6.10	0.002	0.64	0.51/0.81
	Staying in contact with relatives and friends over the phone/internet	3.20	6.80	0.003	0.66	0.53/0.84
	Meeting up with friends in real life	1.60	4.20	0.002	0.61	0.48/0.78
	Staying connected with friends through social media or video games	14.40	8.10	<0.001	1.57	1.18/2.09

OR = odds ratio; CI = confidence interval.

**Table 2:** The proportion of adolescents reporting any change compared to those reporting no change (including those who had never done it) in their behaviours during the COVID-19 pandemic.

		Change		No Change		*p* between Genders
		Women %	Men %	Women %	Men %	
**Risk** **behaviours**	Smoking cigarettes	14.70	15.70	85.30	84.30	0.6
	Consuming alcohol	40.90	41.20	59.10	58.80	0.91
	Getting intoxicated by alcohol	37.10	38.90	62.90	61.10	0.47
	Illicit drug use, including the use of prescription drugs for reasons other than prescribed	5.80	11.20	94.20	88.80	<0.001
	Staying outside/being in the city without the parent’s knowledge	24.80	26.70	75.20	73.30	0.41
	Arguing/fighting with a parent or parents	43.20	33.30	56.80	66.70	<0.001
**Norm-** **breaking** **behaviours**	Stealing from shops/people or from your own or someone else’s home	5.40	5.30	94.60	94.70	0.89
	Harassing someone on the internet using written language or uploaded pictures and/or videos	0.70	1.30	99.30	98.70	0.27
**Salutogenic** **approach**	Spending time with family and taking part in fun activities	66.80	58.80	33.20	41.20	<0.001
	Spending time doing things that one did not have time to do before	54.30	50.60	45.70	49.40	0.15
	Keeping up with school projects and/or work	68.40	66.90	31.60	33.10	0.54
	Having the opportunity to be in control over one’s daily life	69.90	63.30	30.40	36.70	0.01
	Working out or exercising	68.30	68.30	31.70	31.70	0.98
	Being outside and taking walks (for example)	70.40	71.30	29.60	28.70	0.71
	Staying in contact with relatives and friends over the phone/internet	63.10	58.50	36.90	41.50	0.07
	Meeting up with friends in real life	80.60	75.60	19.40	24.40	0.019
	Staying connected with friends through social media or video games	45.40	58.40	54.60	41.60	0.001

## Data Availability

The datasets used and/or analysed during the current study are available from the project leader, Professor Nóra Kerekes, upon reasonable request.

## References

[1] Papadopoulos D. (2023). Impact of Child and Family Factors on Caregivers’ Mental Health and Psychological Distress during the COVID-19 Pandemic in Greece. Children.

[2] Foley S., Badinlou F., Brocki K.C., Frick M.A., Ronchi L., Hughes C. (2021). Family function and child adjustment difficulties in the COVID-19 pandemic: An international study. Int. J. Environ. Res. Public Health.

[3] Shoshani A., Kor A. (2022). The mental health effects of the COVID-19 pandemic on children and adolescents: Risk and protective factors. Psychol. Trauma Theory Res. Pract. Policy.

[4] Pokhrel S., Chhetri R. (2021). A literature review on impact of COVID-19 pandemic on teaching and learning. High. Educ. Future.

[5] Lessard L.M., Puhl R.M. (2021). Adolescent academic worries amid COVID-19 and perspectives on pandemic-related changes in teacher and peer relations. Sch. Psychol..

[6] Tasso A.F., Hisli Sahin N., San Roman G.J. (2021). COVID-19 disruption on college students: Academic and socioemotional implications. Psychol. Trauma Theory Res. Pract. Policy.

[7] Public Health Agency of Sweden Skolbarns hälsovanor I Sverige 2017/18. [Health Behaviour in School-Aged Children (HBSC), Results from Sweden of the 2017/18 WHO Study] 2018. https://www.folkhalsomyndigheten.se/publikationer-och-material/publikationsarkiv/s/skolbarns-halsovanor-i-sverige-2017-2018-grundrapport/.

[8] Larsen B., Luna B. (2018). Adolescence as a neurobiological critical period for the development of higher-order cognition. Neurosci. Biobehav. Rev..

[9] Dahl R.E. (2004). Adolescent brain development: A period of vulnerabilities and opportunities. Keynote address. Ann. N. Y. Acad. Sci..

[10] Golberstein E., Wen H., Miller B.F. (2020). Coronavirus disease 2019 (COVID-19) and mental health for children and adolescents. JAMA Pediatr..

[11] de Miranda D.M., da Silva Athanasio B., Oliveira A.C.S., Simoes-e-Silva A.C. (2020). How is COVID-19 pandemic impacting mental health of children and adolescents?. Int. J. Disaster Risk Reduct..

[12] Viner R., Russell S., Saulle R., Croker H., Stansfield C., Packer J., Minozzi S. (2022). School closures during social lockdown and mental health, health behaviors, and well-being among children and adolescents during the first COVID-19 wave: A systematic review. JAMA Pediatr..

[13] Fegert J.M., Vitiello B., Plener P.L., Clemens V. (2020). Challenges and burden of the Coronavirus 2019 (COVID-19) pandemic for child and adolescent mental health: A narrative review to highlight clinical and research needs in the acute phase and the long return to normality. Child Adolesc. Psychiatry Ment. Health.

[14] Moya-Vergara R., Portilla-Saavedra D., Castillo-Morales K., Espinoza-Tapia R., Sandoval Pastén S. (2023). Prevalence and Risk Factors Associated with Mental Health in Adolescents from Northern Chile in the Context of the COVID-19 Pandemic. J. Clin. Med..

[15] Gardner L.A., Debenham J., Newton N.C., Chapman C., Wylie F.E., Osman B., Teesson M., Champion K.E. (2022). Lifestyle risk behaviours among adolescents: A two-year longitudinal study of the impact of the COVID-19 pandemic. BMJ Open.

[16] Butler N., Quigg Z., Bates R., Jones L., Ashworth E., Gowland S., Jones M. (2022). The Contributing Role of Family, School, and Peer Supportive Relationships in Protecting the Mental Wellbeing of Children and Adolescents. Sch. Ment. Health.

[17] Magson N.R., Freeman J.Y., Rapee R.M., Richardson C.E., Oar E.L., Fardouly J. (2021). Risk and protective factors for prospective changes in adolescent mental health during the COVID-19 pandemic. J. Youth Adolesc..

[18] Okuyama J., Seto S., Fukuda Y., Funakoshi S., Amae S., Onobe J., Izumi S., Ito K., Imamura F. (2021). Mental Health and Physical Activity among Children and Adolescents during the COVID-19 Pandemic. Tohoku J. Exp. Med..

[19] Layman H.M., Thorisdottir I.E., Halldorsdottir T., Sigfusdottir I.D., Allegrante J.P., Kristjansson A.L. (2022). Substance use among youth during the COVID-19 pandemic: A systematic review. Curr. Psychiatry Rep..

[20] Bäker N., Schütz-Wilke J. (2023). Behavioral Changes during the First Year of the COVID-19 Pandemic: A Longitudinal Comparison of Bullying, Cyberbullying, Externalizing Behavior Problems and Prosocial Behavior in Adolescents. COVID.

[21] Garthe R.C., Kim S., Welsh M., Wegmann K., Klingenberg J. (2023). Cyber-victimization and mental health concerns among middle school students before and during the COVID-19 pandemic. J. Youth Adolesc..

[22] Engel G.L. (1977). The need for a new medical model: A challenge for biomedicine. Science.

[23] Public Health Agency of Sweden (n.d.). https://www.folkhalsomyndigheten.se/smittskydd-beredskap/utbrott/aktuella-utbrott/covid-19/folkhalsomyndighetens-roll-under-arbetet-med-covid-19/nar-hande-vad-under-pandemin/.

[24] Andersson F.N., Jonung L. (2024). The COVID-19 Lesson from Sweden: Don’t Lock Down. Econ. Aff..

[25] Worldometers.info (2022). Sweden COVID—Coronavirus Statistics—Worldometer. https://www.worldometers.info/coronavirus/country/sweden/.

[26] Kapetanovic S., Gurdal S., Ander B., Sorbring E. (2021). Reported Changes in Adolescent Psychosocial Functioning during the COVID-19 Outbreak. Adolescents.

[27] Källmen H., Hallgren M. (2024). Mental health problems among adolescents during the COVID-19 pandemic: A repeated cross-sectional study from Sweden. Scand. J. Public Health.

[28] Kerekes N., Bador K., Sfendla A., Belaatar M., Mzadi A.E., Jovic V., Zouini B. (2021). Changes in adolescents’ psychosocial functioning and well-being as a consequence of long-term covid-19 restrictions. Int. J. Environ. Res. Public Health.

[29] (2013). The Swedish National Council for Crime Prevention. https://bra.se/bra-in-english/home/publications/archive/publications/2014-10-30-crime-statistics-2013.html.

[30] de Zarate A.E.R., Thiel A., Sudeck G., Dierkes K., John J.M., Nieß A.M., Gawrilow C. (2023). Well-Being of Adolescents During the COVID-19 Pandemic. Z. Psychologie..

[31] Fan X., Menhas R., Laar R.A. (2023). Repercussions of Pandemic and Preventive Measures on General Well-Being, Psychological Health, Physical Fitness, and Health Behavior: Mediating Role of Coping Behavior. Psychol. Res. Behav. Manag..

[32] Masten A.S. (2010). Ordinary Magic: Lessons from Research or Resilience in Human Development. Educ. Can..

[33] The Organisation of Economical Co-operation and Development (n.d) Sweden. https://www.oecdbetterlifeindex.org/countries/sweden/.

[34] Renati R., Bonfiglio N.S., Rollo D. (2023). Italian University Students’ Resilience during the COVID-19 Lockdown—A Structural Equation Model about the Relationship between Resilience, Emotion Regulation and Well-Being. Eur. J. Investig. Health Psychol. Educ..

[35] Sun B., Mao H., Yin C. (2020). Male and Female Users’ Differences in Online Technology Community Based on Text Mining. Front. Psychol..

[36] Orhon F., Ergin A., Topçu S., Çolak B., Almiş H., Durmaz N., Başkan S. (2023). The role of social support on the relationships between internet use and sleep problems in adolescents during COVID-19 pandemic: A multicentre study. Child Adolesc. Ment. Health.

[37] Deolmi M., Pisani F. (2020). Psychological and psychiatric impact of COVID-19 pandemic among children and adolescents. Acta Bio-Med. Atenei Parm..

[38] McElhaney K.B., Allen J.P., Stephenson J.C., Hare A.L., Lerner R.M., Steinberg L. (2009). Attachment and autonomy during adolescence. Handbook of Adolescent Psychology: Vol 1. Individual Bases of Adolescent Development.

[39] Herke M., Knöchelmann A., Richter M. (2020). Health and well-being of adolescents in different family structures in Germany and the importance of family climate. Int. J. Environ. Res. Public Health.

[40] Moksnes U.K., Innstrand S.T., Lazarewicz M., Espnes G.A. (2023). The Role of Stress Experience and Demographic Factors for Satisfaction with Life in Norwegian Adolescents: Cross-Sectional Trends over a Ten-Year Period. Int. J. Environ. Res. Public Health.

[41] Bera L., Souchon M., Ladsous A., Colin V., Lopez-Castroman J. (2022). Emotional and behavioral impact of the COVID-19 epidemic in adolescents. Curr. Psychiatry Rep..

[42] Nyberg G., Helgadóttir B., Kjellenberg K., Ekblom Ö. (2023). COVID-19 and unfavorable changes in mental health unrelated to changes in physical activity, sedentary time, and health behaviors among Swedish adolescents: A longitudinal study. Front. Public Health.

[43] Hagquist C. (2023). Worry and Psychosomatic Problems among Adolescents in Sweden in the Wake of the COVID-19 Pandemic: Unequal Patterns among Sociodemographic Groups?. J. Adolesc. Health.

[44] Kerekes N., Zouini B., Tingberg S., Erlandsson S. (2021). Psychological Distress, Somatic Complaints, and Their Relation to Negative Psychosocial Factors in a Sample of Swedish High School Students. Front. Public Health.

[45] Sfendla A., Bador K., Paganelli M., Kerekes N. (2022). Swedish High School Students’ Drug and Alcohol Use Habits throughout 2020. Int. J. Environ. Res. Public Health.

[46] Rogés J., Bosque-Prous M., Colom J., Folch C., Barón-Garcia T., González-Casals H., Fernández E., Espelt A. (2021). Consumption of Alcohol, Cannabis, and Tobacco in a Cohort of Adolescents before and during COVID-19 Confinement. Int. J. Environ. Res. Public Health.

[47] Tiburcio N.J., Baker S.L., Kimmell K.S. (2021). Mental Health and Substance Use Disorder Co-Morbidities among Teens in Treatment: SASSI-A3 Correlations in Screening Scores. Psychol. Behav. Sci..

[48] Vallentin-Holbech L., Ewing S.W.F., Thomsen K.R. (2023). Hazardous alcohol use among Danish adolescents during the second wave of COVID-19: Link between alcohol use and social life. Nord. Stud. Alcohol Drugs.

[49] Sudhinaraset M., Wigglesworth C., Takeuchi D.T. (2016). Social and Cultural Contexts of Alcohol Use. Alcohol Res. Curr. Rev..

[50] Westlund O., Öberg J. Strategiska Brott Bland Ungdomar på 2010-Talet—Och Faktorer av Betydelse för att Leva ett Kriminellt liv. [Strategic Crimes among Young People in the 2010s—And Factors of Importance for Living a Criminal Life] (2021:5) 2021. https://bra.se/download/18.1f8c9903175f8b2aa7087f9/1618295581817/2021_5_Strategiska_brott.pdf.

[51] Axell S. (2018). Kortanalys 6/8: Brott i Nära Relationer Bland Unga. [Short Analysis 6/8: Crimes in Close Relationships among Young People]. https://bra.se/download/18.c4ecee2162e20d258c4a9ea/1553612799682/2018_Brott_i_nara_relationer_bland_unga.pdflandunga(bra.se).

[52] Sorrentino A., Sulla F., Santamato M., di Furia M., Toto G.A., Monacis L. (2023). Has the COVID-19 Pandemic Affected Cyberbullying and Cybervictimization Prevalence among Children and Adolescents? A Systematic Review. Int. J. Environ. Res. Public Health.

[53] Westberg S. (2020). Skolundersökning om Brott 2019: Om Utsatthet och Delaktighet i Brott. [School Survey on Crime 2019: About Vulnerability and Participation in Crime]. (2020:11). https://bra.se/download/18.7d27ebd916ea64de5306dead/1606817015507/2020_11_Skolundersokningen_om_brott_2019.pdf.

[54] WMA (2013). Declaration of Helsinki. Ethical Principal för Medical Reseaech. Adopted by the 18th WMA General Assembly, Helsinki, Finland, June 1964 and Amended by the: 64th WMA General Assembly, Fortaleza, Brazil. https://www.wma.net/policies-post/wma-declaration-of-helsinki-ethical-principles-for-medical-research-involving-human-subjects/.

